# Annotations

**Published:** 1903-04-04

**Authors:** 


### Annotations.

### Sewage Effluent Desiderata.

The reports of the Royal Commission on the treatment of sewage are causing some anxiety to sanitary authorities. The last published report is filled with the opinions of experts as to the best methods of getting rid of bacteria from sewage effluents entirely. One gentleman goes so far as to recommend that if by no other method the effluent from sewage works can be rid of these organisms, it ought to be boiled before being permitted to enter rivers. The mere contemplation of the expense of such a scheme is calculated to alarm ratepayers all over the country, and at a recent meeting of the Sanitary Institute Dr. George Reid, Medical Officer of Health for Staffordshire, opened a discussion which had for its object the attempt to prove that a sewage effluent, after proper treatment, may be quite harmless to the fish in the streams into which it is finally discharged and to cattle who may drink the water of these streams, even though it is by no means entirely free from bacilli. With regard to drinking water for human beings, he thought that the onus of providing that should rest with the water companies, and that if, after that, people insisted on using water from the streams into which sewage effluents had been discharged, they must do so at their own peril.

### Tent Life for Consumptives.

The experience of many campaigns and the evidence of not a few in holiday life clearly establish the fact that tent life conduces to the maintenance of a high level of physical vigour. The tent is a habitation of much antiquity, and there is reason to believe that the strength and endurance of many nomadic tribes is directly dependent on the comparatively open-air life which tent accommodation permits. Of this it is certain that the habitations of the so-called civilised are often much inferior hygienically than the home of the savage and the moving camp of the vagabond. The benefit received by consumptives in open-air treatment has focussed attention on the desirability of making suitable dwellings not only for the phthisical and those handicapped by disease but for the growing child and developed adult, in order that nature's powers of resistance to influences making for morbidity should be maintained in full activity. At the present, however, the deeper meaning of the principle of open-air treatment seems in danger of being masked by the elaboration of ideas respecting extensive and expensive sanatoria. Such institutions are no doubt necessary, but amongst many there is danger of an over elaboration which may go far in minimising the benefits which usually follow in the path of simplicity. As a healthy corrective, attention may be drawn to an interesting communication by Dr. C. F. Gardiner, of Colorado Springs, in the last issue of *International Clinics*, on the sanatory tent and its use in the treatment of pulmonary tuberculosis. Dr. Gardiner shows that much of the present day "open-air" treatment is only so in name. He noticed that the Ute Indians in their "tepees" of skin had a tent in which the ventilation was nearly ideal, for there was a hole in the point of the top for the escape of smoke and plenty of space around the lower edge for the entrance of air. Following this hint a tent made of dark brown duck has been constructed with its floor about 8 inches from the ground and with space about its edge for the admission of fresh air and an opening at the top of the tent. Dr. Gardiner, who gives full particulars and plans of his tent, has had it in use under different conditions of climate for several years and found it of great service in dealing with cases which under ordinary sanatorium condition received no distinct benefit. There seems but little doubt that if progress is to be made there must be a limit to the collection of large numbers of cases in huge establishments; and if the cottage system cannot be conveniently employed Dr. Gardiner's suggestion that sanatory tents should be used as a part of a general sanatorium is certainly deserving of careful consideration. An experiment in this direction has recently been carried out at the Mount Vernon Hospital at Hampstead, where, during the reconstruction of a part of the building, many patients were "housed" in a large tent, and we understand with, in most instances, excellent results.

### The Dumb Hour.

To every man death cometh soon or late. In every Life's Day there is a Dumb Hour. It may be in the moments of dawn or when the vitalizing energy is at noon, though sometimes it comes not till the chill and dark of midnight has fallen. There is no more pathetic and yet irresistibly attractive subject for the serious student of medicine than the investigation of the mysteries of that process of physical dissolution which men speak of as dying. And yet there is so personal an interest in the quest that only the bravest minds can grapple undismayed in a research into the problems of Death. We may learn much by a reference to animal instincts in this as in so many other matters. Mr. William J. Long in his recently issued and most charming volume "School of the Woods: Some Life Studies of Animal Instincts and Animal Training" attempts to reveal something of the mystery and pathos of the dumb hour as seen in the dumb beast. In his study on "How the Animals Die" he shows that life's curtain is usually rung down quietly, the footlights are turned out gradually, the auditorium is emptied silently, the darkness deepens peacefully. The animal feels the oncoming of the shadow and creeps into the deepest coverts. The unnumbered multitudes "choose their own place and close their eyes for the last time, as peacefully as ever they lay down to sleep." "The vast majority of animals go away quietly when that time comes; and their death is not recorded because man has eyes only for exceptions. Something calls the creature from his daily round; age or natural disease touches him gently in a way that he has not felt before. He steals away, obeying the old warning instinct of his kind, and picks out a spot where they shall not find him till he is well again." Man thinks himself a little lower than the angels, and it may be so, but of this we may be sure, there is that in the dumb hour which marks the kinship of man with the uncomplaining animal submissive to the decree of nature, and noble in its obedience to the call of the Voice that silences all other voices.

